# RETRACTION: Effect of topical application of autologous platelet gel on sternal wound infection after cardiac surgery: A meta‐analysis

**DOI:** 10.1111/iwj.70080

**Published:** 2024-09-24

## Abstract

Retraction: LiY. , WuZ. , “Effect of Topical Application of Autologous Platelet Gel on Sternal Wound Infection after Cardiac Surgery: A Meta‐Analysis,” International Wound Journal 21, no. 3 (2023): e14761, 10.1111/iwj.14761.PMC1090268838420690

The above article, published online on 29 February 2024, in Wiley Online Library (http://onlinelibrary.wiley.com/), has been retracted by agreement between the journal Editor in Chief, Professor Keith Harding; and John Wiley & Sons, Ltd. It came to the publisher's attention from a third party that a number of articles shared concerning similarities in format and structure. Following an investigation by the publisher, the retraction has been agreed on as the peer review and publishing process for this article were found to be manipulated.

Retraction: Y. Li , Z. Wu , “Effect of Topical Application of Autologous Platelet Gel on Sternal Wound Infection after Cardiac Surgery: A Meta‐Analysis,” International Wound Journal 21, no. 3 (2023): e14761, 10.1111/iwj.14761.PMC1090268838420690

The above article, published online on 29 February 2024, in Wiley Online Library (http://onlinelibrary.wiley.com/), has been retracted by agreement between the journal Editor in Chief, Professor Keith Harding; and John Wiley & Sons, Ltd. It came to the publisher's attention from a third party that a number of articles shared concerning similarities in format and structure. Following an investigation by the publisher, the retraction has been agreed on as the peer review and publishing process for this article were found to be manipulated.

